# *In silico* selection of an aptamer to estrogen receptor alpha using computational docking employing estrogen response elements as aptamer-alike molecules

**DOI:** 10.1038/srep21285

**Published:** 2016-02-22

**Authors:** Rajesh Ahirwar, Smita Nahar, Shikha Aggarwal, Srinivasan Ramachandran, Souvik Maiti, Pradip Nahar

**Affiliations:** 1 Academy of Scientific and Innovative Research, Delhi, India; 2 CSIR- Institute of Genomics and Integrative Biology, Delhi, India

## Abstract

Aptamers, the chemical-antibody substitute to conventional antibodies, are primarily discovered through SELEX technology involving multi-round selections and enrichment. Circumventing conventional methodology, here we report an *in silico* selection of aptamers to estrogen receptor alpha (ERα) using RNA analogs of human estrogen response elements (EREs). The inverted repeat nature of ERE and the ability to form stable hairpins were used as criteria to obtain aptamer-alike sequences. Near-native RNA analogs of selected single stranded EREs were modelled and their likelihood to emerge as ERα aptamer was examined using AutoDock Vina, HADDOCK and PatchDock docking. These *in silico* predictions were validated by measuring the thermodynamic parameters of ERα -RNA interactions using isothermal titration calorimetry. Based on the *in silico* and *in vitro* results, we selected a candidate RNA (ERaptR4; 5′-GGGGUCAAGGUGACCCC-3′) having a binding constant (*K*a) of 1.02 ± 0.1 × 10^8 ^M^−1^ as an ERα-aptamer. Target-specificity of the selected ERaptR4 aptamer was confirmed through cytochemistry and solid-phase immunoassays. Furthermore, stability analyses identified ERaptR4 resistant to serum and RNase A degradation in presence of ERα. Taken together, an efficient ERα-RNA aptamer is identified using a non-SELEX procedure of aptamer selection. The high-affinity and specificity can be utilized in detection of ERα in breast cancer and related diseases.

Human estrogen receptor α (ERα), a 66 kDa ligand- inducible transcription factor is a key mediator of 17β-estradiol induced proliferation, differentiation and development of breast and uterine tissues. ERα is a crucial biomarker useful in breast cancer diagnosis and treatment^1^,^2^. Presence of ERα in almost two-thirds of tumours and their subsequent treatment towards regression with hormonal therapy has established ERα as a useful target for clinical purposes^3^,^4^,^5^,^6^. Detection of the altered expression of ERα in breast cancer and related diseases is carried out using ERα antibodies. However, the antibody- based applications are usually fraught with high costs and complexity of production, batch to batch variability, cross-reactivity, contamination, and short shelf life^7^,^8^,^9^. The substitute to conventional antibodies could be met by next generation affinity molecules that overcome the limitations of cost, synthesis, stability and specificity of target-binding^9^. Aptamers have gained considerable importance as selective and affinity-binding molecules due to the fact that the need for animals is obviated in their production with the added advantage of reduced time and production cost. Aptamers are short oligonucleotides that can be raised against almost every molecule^10^,^11^. They offer great advantages due to their high target specificity, affinity, low molecular weights and the usual non-immunogenic nature^12^. Aptamers are mostly identified through an iterative process called systematic evolution of ligands by exponential enrichment (SELEX), which is a cyclic process that involves multiple rounds of selection and amplification^11^. The entire process is tedious, time consuming and often fails to enrich high affinity aptamers^13^. Additionally, the requirement of fixed priming sites in the sequences of a library imposes a length criterion on random region, thereby restrict the diversity of the synthesized aptamer library. Even the lengthy aptamers, usually ≥40 nt long require prior trimming and shortening for their efficient scale-up production. Recently, ER targeting aptamers have been identified using classical SELEX screening employing multiple rounds of protein-aptamer binding, selection, amplification and enrichment^14^. However, these aptamers were too lengthy and required truncations to obtain smaller aptamers. In a similar approach, He X. *et al.,* reported the use of conditioned library for a selection of the ER-aptamer, but they still required conventional SELEX for aptamer screening^15^. Bioinformatic approaches that can combine *in vitro* and *in silico* methodologies could provide better solutions for aptamer screening and selection^16^,^17^. Methodologies such as DNA/RNA microarray provide high throughput mean to isolate aptamers, but at the same time, are limited by the requirement of pre-selected pool of minimal sequence numbers (~10^4^–10^5^) for chip synthesis. Also, the method that relies on a specifically designed RNA pool for aptamer selection against specific-analytes can be restricted by requirement of heavy computation and time^16^.

To identify an ERα-targeting aptamer by a non-SELEX procedure, we hypothesize that the estrogen response elements, which are the stretches of B-DNA in the promoter region of the genes regulated by estrogen receptors^18^,^19^, can be utilized to obtain a pool of aptamer-alike sequences for *in silico* screening. Their inverted repeat nature and potential to interact with ERα *in vivo* provides a way to mimic these characteristics in an *in silico* system. Virtually the single strands of the EREs having inverted repeats can adapt stable hairpins and may act as potential aptamers. To prove this, we have developed a non-SELEX method that combines the *in vivo* chemistry of ERE structure and interactions with computation modelling and molecular docking to identify an ERα binding aptamer. Accordingly, a selection criterion is drawn to obtain hairpin-forming EREs and their RNA analogs are modelled to analyze their binding with ERα using AutoDock Vina, HADDOCK and PatchDock docking^20^,^21^,^22^. These *in silico* predictions are further validated by *in vitro* affinity measurement. A candidate sequence is selected as ER-aptamer and evaluated for its target- specificity. Also, solid-phase assays are performed to demonstrate the antibody-alternative action of the selected ERα-aptamer.

## Results

### Structural and functional selection of EREs and their 3D structure modelling

**Figure 1:**
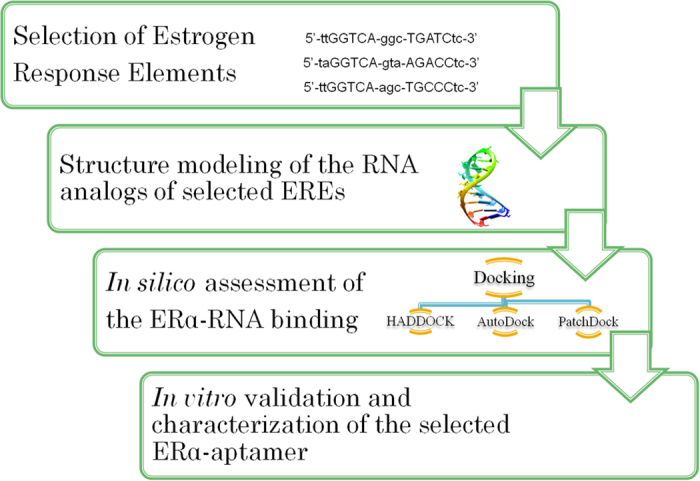
Flow chart showing the designed *in silico* approach of non-SELEX selection of an ERα binding aptamer.

ERs execute the expression of target genes either by binding directly to EREs or by associating and recruiting transcriptional machinery at the promoter sites of ER target genes^23^. Many genes in human have been identified to contain ERE in their promoter proximal regions, where the ER complex binds and initiates the transcription of target genes. We have used these specialized ERE-ER interactions as a model towards developing ERα binding RNA aptamers. The fact that the ERs interact directly to ERE have offered us to model sequences analogous to EREs. In addition, we aimed to preserve the inverted repeat structure of the modelled sequence similar to the natural EREs. Therefore, a selection criterion was drawn to select human EREs which are full length inverted repeats and also involve in direct binding with ER *in vivo*. The prerequisite of full-length palindromes was to ensure the selected sequences to adopt stable hairpin loops. At the same time, the requirement of *in vivo* interacting partners was made to capitalize the inherited binding inclination of ERs for EREs. As a result of the designed selection criterion, we obtained eighteen EREs that matched our criteria (Table 1). These selected EREs are present mainly in the genes that code for catalytic proteins, the proteins of the immune system, hormones, growth factors, and proteins that are involved in cancer initiation and progression. As listed in Table 1, we select the sense strands of these EREs and model their RNA analogs using MC-Fold and MC-Sym algorithms^24^. For the majority of the modelled RNAs, the average number of generated tertiary structures ranged between five to hundred. We observed that the number of generated tertiary structures was more for sequences forming moderately stable hairpins, whereas these numbers remained confined to a few for sequences that form strong hairpins. We only selected structures having the lowest free energy (near native structure) for each of the modelled RNA. As depicted in Fig. 1, these modelled RNAs were used as ligand in the subsequent docking experiments.

### Virtual screening to identify probable aptamers for ERα

To identify the ability of modelled RNA sequences to emerge as ERα aptamer, we designed an *in silico* approach that used these RNAs as ligand to predict their binding with ERα. For this binding prediction, we used three different docking platforms, namely the AutoDock Vina, HADDOCK and PatchDock.

The designed *in silico* approach was first tested on a set of putative aptamer-protein complexes to evaluate its target-specific predictions. Thrombin-RNA aptamer complex^25^ and VEGF_165_-RNA aptamer complex^26^ were selected as test complexes. PDB coordinates of thrombin (1PPB) and VEGF_165_ (2VGH), and modelled aptamers (Thrombin: 5′-gggaacaaagcugaaguacuuaccc-3′; VEGF_165_: 5′-ccgguagucgcauggcccaucgcgcccgg-3′) were used as input in AutoDock Vina, HADDOCK and PatchDock docking. In parallel, the efficacy of the *in silico* approach was tested by performing control docking using similar length random RNA sequences (Table S1). The obtained docking scores from all the three docking algorithms were normalized to an arbitrary unit using mean centered Z score as calculated using equation^27^:


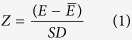


where E is the obtained binding score of individual RNA- protein complex (in a set of 10 best binding modes), Ē is the mean binding score and SD is the standard deviation. Most negative Z-score in a set of 10 best binding modes of an RNA-protein complex was taken as docking specific Z-score of that particular complex. Total Z-score (Z_T_) was computed by adding the Z-scores of HADDOCK (Z_H_), PatchDock (Z_P_) and AutoDock Vina (Z_AV_). We obtained a Z_T_ value of −5.6 and −5.0 for thrombin-RNA aptamer complex and VEGF_165_-RNA aptamer complex, respectively (Supplementary Table 1). Contrary to this, the Z_T_ scores of random-RNAs complexed with the thrombin (mean = −3.62, SD = −0.29, t(5) = 8.97, p value = 0.0003) and VEGF_165_ (mean = −3.40, SD = −0.25, t(5) = 8.38, p value = 0.0004) were found to be significantly low. This suggests that the designed *in silico* approach of aptamer selection can selectively predict and differentiate among target-specific and non-specific binding partners in a DNA-protein complex.

**Figure 2:**
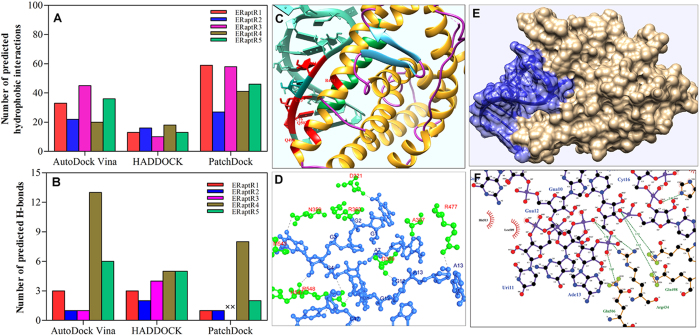
Analysis of the predicted intermolecular interactions in the selected ERα-RNA complex. (**A,B**) Numbers of the predicted hydrophobic interactions and H-bonds in complex of ERα with ERaptR1-ERaptR5. These interactions are predicted using Ligplot and Nucplot. (**C**) Ribbon view of the HADDOCK predicted ERα (1SJ0)-ERaptR4 complex, depicting the interacting residues and the spatial arrangement of protein chains in the vicinity of aptamer molecule. (**D**) H-bonding residues in the AutoDock Vina generated complex of ERα-ERaptR4. The blue colour represents the aptamer bases while the green colour indicates the amino acids. (**E**) Surface view of the PatchDock generated ERα-ERaptR4 complex showing the relative orientations of interacting bases and amino acid chain. (**F**) Structural representation of H-bond and hydrophobic interactions in the ERα-ERaptR4 complex as predicted using Ligplot. H-bonds are represented by dashed lines between H-bonding atoms, whereas the hydrophobic interactions are shown by an arc with spokes radiating towards the interacting ligand atoms.

After the method testing, we predicted binding partners for ERα (1SJ0) using the selected RNA sequences as ligands. Eighteen docking experiments, using AutoDock Vina, HADDOCK and PatchDock docking algorithms were carried out to predict the most probable aptamer in the collected set of sequences. The strength of the binding interactions in resulting complexes was estimated using Z_T_ scores (Table 2). Further, control docking were performed using hairpin-forming random RNA sequences to ascertain the specificity in the predictions (Supplementary Table 2). The variance in the Z_T_ scores of ERα -RNAs (ERaptR1-ERaptR18) and ERα-random RNAs complexes were measured using *t*-test analysis. The results showed a statistically significant difference in the Z_T_ values of random RNAs (mean = −3.56, SD = 0.51, n = 5) and RNA analogs of ERE (mean = −4.44, SD- = 0.76, n = 18, t(21) = 2.73, p value = 0.0273). Further, this difference in Z_T_ score got more pronounced when we analyzed the predicted binding of random RNAs with only the top five candidates from the Z_T_ sorted RNAs (mean = −5.19, SD = 0.36, n = 5, t(8) = 5.75, p value = 0.0004). This suggested that not all RNA hairpins can form stable complexes with ER. Also, this indicates that the ERα-RNA complexes with highest Z_T_ scores may better represent the near-native complexes, and emerge as promising aptamer to ERα. To test this assumption, we further analyzed the role of hydrogen bonds and hydrophobic interactions in these predicted aptamer-protein complexes^28^. We estimated the strength of binding in the selected ER-RNA complexes by measuring the intermolecular H-bonds and hydrophobic interactions (Fig. 2). Although, none of the predicted complexes showed an overall advantage of hydrophobic interactions, the aptamer-protein complex of ERaptR4 (predicted by HADDOCK, PatchDock and AutoDock Vina) was found to form the maximum number of intermolecular H-bonds with the ER protein (Fig. 2). The detailed description of the predicted interacting (partner) atoms is provided in supporting information (Table S3).

Also, as the selected RNA sequences form stable hairpins, we assumed that the free energy of secondary structure formation might play some role in deciding the binding energetics of the ERα-RNA complex. Accordingly, we predicted the free energy (Δ*G*) of secondary structure formation for selected probable aptamers and compared them with the mean free energy of the control RNAs that form similar hairpin structures (Table 3). However, we found no significant difference in the predicted Δ*G* value of probable aptamers (M = −6.96, SD = 2.61) and the random RNAs (M = −3.68, SD = 2.78; t(8) = 1.92, p-value = 0.0911). This further supports our previous prediction that irrespective of similar hairpin formation, the selected probable aptamers form an energetically stable complex with ERα.

Taken together, the *in silico* predictions (ZT score and H-bond interaction) suggests that the selected RNA analogs of the ERE can emerge as aptamers to the ERα. Moreover, the Z_T_ sorted top five candidates (ERaptR1-ERaptR5) holds higher potential to act as prominent ERα-aptamer.

### *In silico* predicted RNA candidates emerged as high affinity and specific aptamers to ERα

**Figure 3:**
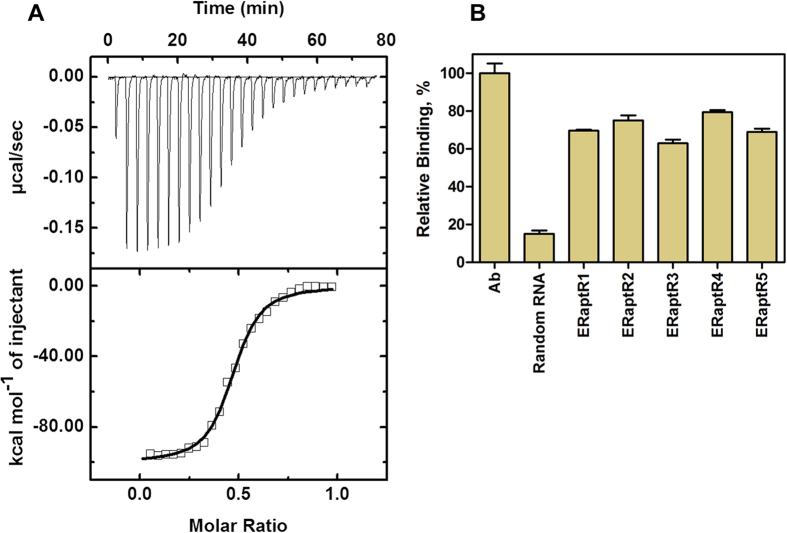
Measuring the *in vitro* affinities of the *in silico* selected ERα-aptamers. (**A**) ITC isotherms of ERα interactions with aptamer ERaptR4. For each titration, the ERα concentration in 1.4 ml sample cell was taken as 1 μM and aptamer concentration in syringe was 10 μM. The top panel represents the raw heats of binding obtained upon titration of aptamer to ERα protein. The lower panel is the binding isotherm fitted to the raw data using one site model. (**B**) ELISA-based measurement of the relative binding of selected sequences with ERα. Binding of aptamer candidates is presented after normalizing against the ERα-antibody control. A random 17-mer RNA sequence (5′-aucgugugcugcuacga-3′) is taken as a random RNA control.

Evaluation of the *in silico* predictions was carried out by measuring the binding affinities of all the probable aptamers (RNAs) with ERα using isothermal titration calorimetry. A hairpin forming random RNA was used as a random control. As summarized in Table 4 (also Fig S1–S4), the majority of the selected RNAs showed a preferential binding to the ER with the values of binding constant (*K*a) of an order of 10^7 ^M^−1^. However, we obtained no detectable binding between selected random RNA sequence and the ERα. Interestingly, the thermodynamic parameters (Δ*H* and Δ*G*) of ERaptR4 binding to ER were found to be most favoured for its selection as an RNA aptamer to ERα (Fig. 3A and Table 4). Although the ERaptR17 also showed similar binding, we only tested the ERaptR4 as a candidate aptamer and performed investigations to analyze its specificity for ERα.

Nevertheless, we also performed aptamer-assisted ELISA^29^ to analyze the binding characteristics of selected sequences in solid-phase assays. The relative binding of the top five RNAs (ERaptR1-ERaptR5) with ERα was assessed with an ERα-antibody control. As depicted in Fig. 3B, the ERaptR4 showed a relatively better binding to ERα. These results were in agreement with the *in silico* predictions and thermodynamic measurements.

Altogether, the predicted Z_T_ score, H-bond interactions, and the measured value (*K*a) of ER-ERaptR4 binding confirm the likelihood of ERaptR4 as a promising aptamer to ERα. The observed differences in the predicted order (ZT-sorted) of aptamers and the calculated values of binding constants could be attributed to the mechanistic differences in the *in silico* and *in vitro* systems. Nevertheless, the occurrence of an ERα aptamer in the selected EREs provides a measure of the feasibility of the present method in identifying aptamers in a non-SELEX manner.

### ERaptR4 binding to ERα is highly selective

**Figure 4:**
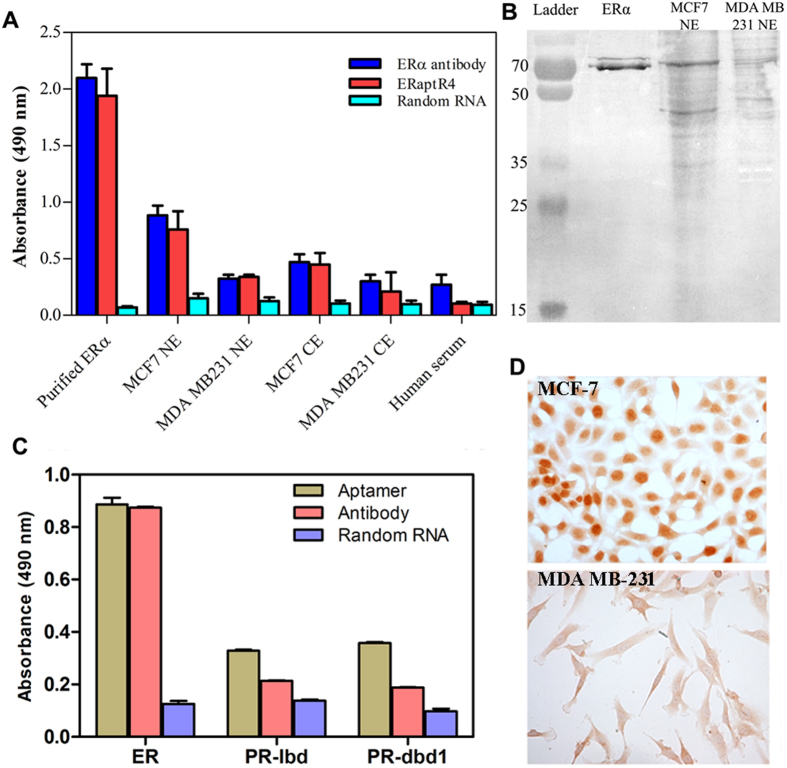
Validating the target specificity of ERaptR4 aptamer. (**A**) The specificity of ERaptR4 binding to ERα, as estimated by an ELISA-based detection of purified ERα, nuclear and cytoplasmic extracts of MCF-7 and MDA MB-231 cells and human serum proteins, using biotinylated ERaptR4 as detection molecule. A 17-mer random RNA sequence (5′-aucgugugcugcuacga-3′) was used as random RNA control. Data is plotted after subtracting the background binding. (**B**) Western blot analysis of SDS PAGE separated ERα, MCF-7 and MDA MB-231 nuclear extract using biotinylated ERaptR4. (**C**) ELISA-detection of ER (lbd) and PR (lbd and dbd) using biotinylated ERaptR4. Random RNA and ER-antibody were taken as negative and positive controls, respectively. (**D**) Cytochemical detection of ERα in the fixed monolayer culture of MCF-7 and MDA MB-231 breast cancer cells as carried out using biotinylated-ERaptR4.

As the specificity of aptamers is an important aspect of their biological action, we evaluated the target specificity of the ERaptR4 in various solid phase assays. Aptamer and antibody- ELISA was carried out for detection of ERα in different samples varying in the complexity and availability of target-ERα. As depicted in Fig. 4A, the aptamer was found to produce an antibody-equivalent signal in almost all the analyzed samples. The aptamer showed no binding with the cellular extracts of ERα-deficient MDA MB-231 cells, but produced an excellent signal in the nuclear and cytoplasmic extracts of ERα-positive MCF-7 cells. This shows that the aptamer can specifically target the ERα without any cross-reactivity to non-target components.

These observations were further confirmed through ERaptR4-assisted western blot assay, wherein the SDS PAGE separated samples of ERα were detected using biotinylated ERaptR4 instead of an ERα-antibody. As shown in Fig. 4B, the presence discrete bands at 66 kDa (full length ERα) and 46 kDa (transcript variant) in purified -ERα and MCF-7 nuclear extract samples support the specific binding of ERaptR4 to ERα. Absence of such discrete bands in the extracts of MDA MB-231 breast cancer cells suggests the lack of cross-reactivity. Further, we checked the cross reactivity of ERaptR4 against the progesterone receptor. As shown in Fig. 4C, we found an insignificant binding of ERaptR4 to the DNA binding and ligand binding domains of PR. Despite of considerable homology in the ER and PR, the lack of ER aptamer-binding to PR suggests the high target specificity of the *in silico* selected aptamer.

Further validation of the affinity and specificity of ERaptR4 for ERα was carried out using chromogenic cytochemistry performed on MCF-7 and MDA MB-231 breast cancer cells. The formalin-fixed monolayer culture of these cells were stained with biotinylated ERaptR4 and visualized under microscope. As depicted in Fig. 4D, the ERaptR4 bind selectively to the ERα present in the nuclear region of MCF-7 cells, without cross reacting to other cellular/extracellular components. Also, the ERaptR4 showed no binding to any of the cellular components of ERα-deficient MDA MB-231 cells. Furthermore, these observations suggest the diagnostic applicability of ERaptR4 in detecting the ERα in breast cancer or related diseases.

### ERaptR4 can sustain nuclease and serum degradation in presence of ERα

**Figure 5:**
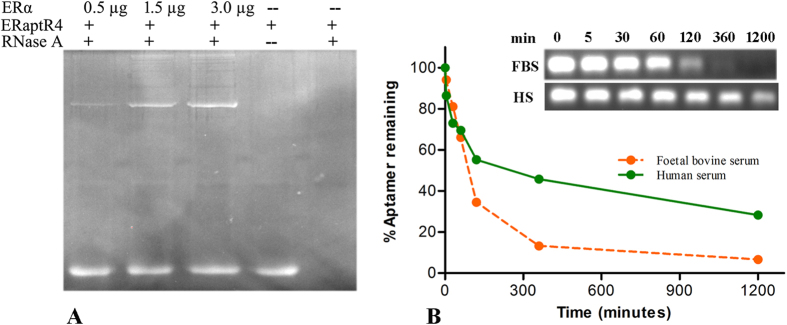
Stability analysis of ERaptR4. (**A**) Nuclease stability of ERaptR4, as measured using the RNA protection assay. ERaptR4 (2.0 μg) was incubated with RNase A in the presence of 0.5, 1.5 and 3.0 μg of ERα. The samples were separated on 2.0%. The un-degraded ERaptR4 was detected using EtBr staining. (**B**) Serum stability of ERaptR4 was examined in 10% foetal calf serum and human serum for time intervals of 0–1200 minutes. The un-degraded ERaptR4 was determined by separating it on 2% agarose and EtBr staining. The graph was normalized by taking the fluorescence intensity of initial sample (0 min) as 100%.

Shelf life and nuclease stability are two important parameters that can decide the clinical and research utility of an aptamer. Towards this, we have analyzed the stability of ERaptR4 against the RNase and serum degradation. The target-dependent stability of aptamer against RNase A was tested using the classical RNA protection assay. As shown in Fig. 5A, ERaptR4 showed complete rescue from nuclease digestion in the presence of its target protein. However, in absence of ERα, the aptamer is susceptible to nuclease degradation. Thus, the diagnostic assays that will involve the ERα would be least hindered by the problem of nuclease digestion, as the ER will act as a mask to the aptamer. Also, we measured the stability of ERaptR4 aptamer in 10% foetal bovine and human (female) serum. We observed that ERaptR4 undergoes a time dependent degradation in both the serum samples; the rate of aptamer degradation was faster in foetal bovine serum with an approx. half life of 100 minutes. However, the same aptamer has resisted the degradation in human serum as indicated by the approx. half life of 240 minutesFig. 5B. The conditions of the hosts during the time of serum isolation and the difference in their nucleases profile could have accounted for such observations. However, for the majority of the detection assays, a serum half life of 100 min can yield sufficient results without compromising the specificity or affinity of the method.

## Discussion

Selection of aptamers using the classical Systematic Evolution of Ligands by Exponential Enrichment (SELEX) method has some inevitable shortcomings that limit its efficacy to produce high affinity and selective-aptamers in a reasonable time and with limited resources. The SELEX process needs repetitive selection and enrichment, optimally 10–20 to enrich an aptamer. This makes the selection process to last for weeks to months. Similarly, the use of priming regions in the sequences of an aptamer library can hinder the aptamer-target interactions. This can even result in enrichment of low affinity binders. Also, the constant regions at the end of each sequence restrict the diversity of synthesized libraries or requires synthesis of longer libraries to achieve an adequately diverse pool. As an alternate, methodologies such as microarray, high throughput sequencing or computation-assisted SELEX have been reported to ease the process of aptamer selection. However, the requirement of the lesser population of sequences (e.g. 10^4^-10^5^) for microarray and the heavy computations in reported *in silico* methods limit the effective utilization of the alternate methods^30^,^31^,^32^,^33^.

The present study overcomes some of these limitations by reporting a non-SELEX method of ERα-aptamer selection. We applied the novel concept of using selective single stranded EREs as a probable pool of ERα-specific aptamers. Though the *in vivo* binding of dimerized estrogen receptors at double stranded ERE of target genes have been known for decades and many studies have reported these interactions in an *in vitro* system as well^18^, there is hardly any report on the binding of a single stranded ERE to a monomeric ER. In the single strand conformation, these EREs can adopt stable hairpins because of their inverted repeat nature. Further, they retain their inherent binding for ER as we selected only full length EREs. On the basis of these, we reasoned that hairpin forming full-length EREs can mimic structures akin to the aptamers. As the number of known EREs is vast, this required a selection procedure to obtain such aptamer-alike sequence. We capitalized the aptamer-likeness of EREs by selecting full length palindromic EREs that can form stable hairpins. Interestingly, as the criterion of ERE selection is dependent on their structural makeup, a similar approach can also be utilized to obtain hormone response elements as probable aptamer pool for other nuclear receptors. This also makes our approach a method of choice for non-SELEX selection of aptamer for various nuclear receptor transcription factors.

Further, as RNA aptamers are known to possess higher structural and functional adaptability and prove more pivotal than DNA aptamers^34^,^35^, we converted the selected single strands of EREs from their DNA to RNA analogs to avail the RNA-associated benefits. These RNAs were used as ligand in a docking approach that used three docking algorithms to capture a wide spectrum of docking predictions. AutoDock Vina is a flexible docking algorithm that uses Monte Carlo simulated dockings to predict the native binding mode of protein-RNA complex. HADDOCK (High Ambiguity Driven DOCKing) is a data driven method that predicts minimal energy conformations from experimentally or bioinformatically available interaction information. Unlike the two, the PatchDock is a geometry-based rigid-body docking that provides complexes which are sorted by molecular shape complementarity criteria. The approach of using three docking algorithms to predict the binding of an RNA-protein complex was tested on pre-defined molecules and found to selectively differentiate in to specific and non-specific binding partners of a protein. Using this approach, we have shown that hairpins forming EREs can be raised as ERα aptamer. Concomitant statistical analyses were done to prove the significance of the *in silico* predictions.

We further validated these *in silico* predictions by measuring the *in vitro* affinity and specificity of selected sequences. With the help of isothermal titration calorimetry, we have shown that the RNA analogs of ERE that we initially obtained as a pool of probable ERα-aptamers are indeed the true binding partners of ERα. All the selected RNAs showed a preferential and high affinity binding for ERα. The lack of binding between ERα and a random RNA (hairpin) provided the measure of the target specificity of selected probable aptamers. Further, a stoichiometry of 0.5 for RNA-ERα binding is indicative of the presence of two binding sites (corresponding to each half ERE) on selected RNAs analogs of the EREs. Nevertheless, the docking -predictions has made it easy to shortlist the most prominent aptamer among selected RNAs. The *in silico* anticipation of the maximum hydrogen bonds and the favouring thermodynamics of the ERα-ERaptR4 interaction at 25 °C (Δ*G* = −11.1 kcal/mol; *K*a = 1.02 ± 0.1 × 10^8 ^M^−1^) are the indicative of high affinity and selective binding of ERaptR4 to ERα.

As the conventional method of ER detection mostly uses antibodies; by substituting the ER-antibody by ERaptR4 in assays such as the aptamer-assisted ELISA, western blot, and cell-imaging, we have shown that the ERaptR4 can efficiently alternate the ERα-antibodies in these immunoassays. These successful attempts to specifically detect and quantify ERα in solid- or solution-phase assays provided additional evidence on target- specificity of the selected ERα aptamer. Further, as the proteins of the nuclear receptor family share conserved domains among various receptors, we checked the cross reactivity of ER-aptamer with the progesterone receptor. The lack of binding to either the LBD or DBD of PR promises high target specificity and concomitant applicability of the ERaptR4.

In conclusion, our study provides an RNA aptamer to ERα, selected through a non-SELEX *in silico* method of aptamer selection. The single stranded estrogen response elements were used as a pool of probable aptamers and their aptamer-likeness was predicted using computational dockings. As the developed *in silico* method SELEX-free aptamer selection is cost effective, simple and does not require sophisticated instruments, it can be applied to obtain aptamer against other nuclear receptors.

## Methods

### Selection of ERE sequences and their tertiary structure predictions

Putative EREs were screened according to the designed criteria. EREs with extended half-site were removed and not included in the selection. Sense strands of selected 17 EREs that matched our criteria and a sequence form solved crystal structure of ERα-DNA complex^36^ were chosen for tertiary structure modelling using MC-Fold and MC-Sym algorithm (http://www.major.iric.ca) to obtain tertiary RNA analogs of selected EREs. MC-Fold MC-Sym algorithms work in a pipeline mode, where the MC-Fold generate secondary structures for each input RNA sequence and these structures are used as input sequences by MC-Sym to model all possible tertiary structures. Minimum free energy structures corresponding to each input sequence were selected and used as ligands in subsequent *in silico* docking analysis.

### Virtual screenings

The PDB coordinates of ER LBD (1SJ0, resolution 2.03 Å) were taken from Protein Data Bank and prepared for dockings by deleting undesired protein chains and ligand. For AutoDock Vina, the grid box was specified in the coordinate system of ER corresponding to BindN+^37^ predicted RNA-binding amino acids. Grid box was created with a default value of 0.375 Å for spacing between the grid points centering at 11.139, 10.861, 9.611 and 60, 62, 68 points in x, y and z dimensions, respectively. Docking was carried out at HPC environment. Vina generates a single pdbqt file containing top ranked binding modes of minimum free energy conformation upon successful completion of docking. HADDOCK dockings were performed on easy interface of the server using already prepared receptor and ligand files. HADDOCK uses ambiguous interaction restraints to run the docking process repetitively, to insure generation of the highest number of correct decoys. Further, any decoys which are driven by wrong restraints were discriminated based on their lower scores compared to the correct decoys. Output decoys are provided as water-refined structure sorted by its HADDOCK-score. Molecular shape complementarity docking was performed over PatchDock web server. Prepared PDB files of ligand and receptor was provided to PatchDock server at default value of 4.0 for clustering RMSD and default complex type. PatchDock represents the Connolly’s surface of docking partners as concave, convex and flat patches and matches them to generate candidate transformations. The PatchDock-generated transformations (protein-RNA complex) were further refined by FireDock^38^ to obtain best transformations from each dockings. In total, 18 independent docking runs were performed with each docking algorithm to evaluate the ERα binding potential of individual RNA analog.

### Isothermal titration calorimetry

Dissociation constant was measured using isothermal titration calorimetry experiment performed at 25 °C using a MicroCal VP-ITC (MicroCal, Inc., Northampton, MA, USA). ERα and aptamer solutions were prepared in 10 mM Tris-HCl, pH 8.0. The thermal equilibration step at 25 °C was followed by an initial 120 s delay step and the subsequent twenty five injections of 10 μM ERaptR4,injected at 370 rpm to 1.0 μM of ERα protein (injection duration of 10 s and spacing of 180 s). Each injection generated a heat-burst curve between micro cal s−1 versus time (min). The saturation curve between kcal/mol of injectant vs. molar ratio was determined by integration, using Origin 7.0 software (Microcal, Inc.) to give the measure of the heat associated with the injection. The resulting experimental binding isotherm was corrected for the effect of titrating estrogen receptor alpha to its binding buffer. The binding affinity and thermodynamic parameters of the binding process were obtained by fitting the integrated heats of binding the isotherm to the one site binding model to give a association constant (*K*a), stoichiometry (n) and the binding enthalpy and entropy (Δ*H*, Δ*S*). The Gibbs free energy (∆*G*) was calculated using the equation^39^:


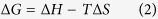


### Aptamer-assisted ELISA

Aptamer-assisted ELISA was carried out in FNAB activated microtiter plates carrying equivalent amount of immobilized ERα, the nuclear and cytoplasmic extracts of MCF-7 and MDA MB-231 breast cancer cells, and human serum proteins. The wells were loaded with 100 nM biotinylated ERaptR4, incubated for 2 h and followed by detection of target bound aptamer using streptavidin-HPR conjugate. Specificity was estimated using the measured absorbance values against individual targets. In parallel, ERα antibody and a 17-mer random RNA sequence was used as positive and negative controls, respectively.

### Aptamer-assisted Western blot

Target samples were separated on 12% SDS PAGE and electroblotted to PVDF membrane. The membrane was then coated with 5% BSA, and afterwards incubated with 1 μM ERaptR4. Development of the blot was carried out using streptavidin-HRP conjugate.

### Aptamer-assisted cytochemistry assay

Cytochemistry assay was performed on p-formaldehyde fixed monolayer cultures of MCF-7 and MDA MB-231 cells. The fixed cells were permeabilized by 0.1% Triton X-100 (in PBS) by 10 min incubation at RT. Cells were blocked with 1% BSA and 22.5 mg/ml glycine for 30 min and afterward incubated with 200 nM of biotinylated ERaptR4 for additional 2 hours. This followed PBS wash and subsequent incubation with streptavidin-HRP conjugate (1:500 dilution) at RT for 1 hour. Cover slip coated cells were then stained with DAB staining solution (500 μL 1% DAB in 5 mL 1X PBS, 15 μL H_2_O_2_) and counterstained with haematoxylin. After mounting with anti-fade mounting solution, the images of stained cells were taken using Nikon Eclipse i9 microscope.

### Serum stability measurement

Serum stability of ERaptR4 was examined in 10% FBS and 10% human female serum. For this, 2 μg of ERaptR4 in 10 μL of respective serums was incubated for 0–20 h. Samples were collected at stipulated time periods and frozen immediately to −70 °C until analyzed on 2% agarose gels.

### RNase A digestion assay

RNase A digestion assay was performed by incubating 0.5 μg, 1.5 μg and 3.0 μg of ERα with 10 μM of ERaptR4. The binding was allowed for 30 min and then incubated with 100 μg/mL of RNase A at 37 °C for 5 minutes. The reaction was terminated by heating the sample at 85 °C for 10 min, followed by treatment with proteinase K (20 mg). The sample was then loaded on a 2% agarose gel in 1X TAE and run at 60V for 30 min and visualized by EtBr staining.

## Additional Information

**How to cite this article**: Ahirwar, R. *et al.* *In silico* selection of an aptamer to estrogen receptor alpha using computational docking employing estrogen response elements as aptamer-alike molecules. *Sci. Rep.* **6**, 21285; doi: 10.1038/srep21285 (2016).

## Supplementary Material

Supplementary Information

## Figures and Tables

**Table 1:** List of the target genes and corresponding single-stranded estrogen response element sequences used for RNA modeling and aptamer predictions.

Target gene description	Target ERE sequence	Reference
Telomerase reverse transcriptase	TTGGTCAGGCTGATCTC	40
Trefoil factor 1 (pS2)	AAGGTCACGGTGGCCAC	41
Keratin-19	TAGGTCAGTAAGACCTC	42
Oxytocin	GGGGTCAAGGTCACCGC	43
Hageman Factor XII	TTGGTCAAGCTGCCCTC	44
Complement C3	AGGGTCAGGGCCACCTG	45
Lactotransferrin	CAGGTCAAGGCGATCTT	46
Angiotensin	CGGGTCACGATGCCCTA	47
Transforming growth factor, alpha†	GCGGTCACCGTCACCTC	48,49
Transforming growth factor, alpha††	GGGGTCAGCTGTGCCCCG	48,49
Vascular endothelial growth factor	CCAGTCAGTCTGATTAT	50
Lipocalin-2	GAGGTCACTGAGACCAT	51
Cathepsin D	CCGGTCACGTGGGCGCG	52
Estrogen-Responsive Finger Protein	AGGGTCATGGTGACCCT	53
Cytochrome c oxidase subunit VIIa related protein	GGGGTCAAGGTGACCCC	54
Estrogen receptor binding site associated antigen 9	CGGGTCAGGGTGACCTC	54,55
Human genome Alu ERE	CAGGTCAAGGCGATCTT	56
Solved crystal structure	CAGGTCACAGTGACCTG	36

^†^Location of estrogen responsive region −252 to −200.

^††^Location of estrogen responsive region −1527 to −1511.

**Table 2:** Z-score values of docking predicted ERα-RNA complexes.

Probable aptamers	Sequences of RNA analogs of aptamer-alike EREs†	AutoDock Vina Z-score (Z_AV_)	Haddock Z-score (Z_H_)	PatchDock Z-score (Z_P_)	Total Z-score (Z_T_)
ERaptR1	GAGGUCACUGAGACCAU	−2.04	−2.05	−1.62	−5.70
ERaptR2	CCAGGUCACAGUGACCUG	−1.56	−1.89	−1.87	−5.32
ERaptR3	AGGGUCAGGGCCACCUG	−2.21	−1.53	−1.54	−5.28
ERaptR4	GGGGUCAAGGUGACCCC	−1.76	−1.87	−1.27	−4.90
ERaptR5	AAGGUCACGGUGGCCAC	−1.36	−1.98	−1.45	−4.79
ERaptR6	UAGGUCAGUAAGACCUC	−1.89	−2.31	−0.57	−4.77
ERaptR7	CGGGUCACGAUGCCCUA	−1.90	−1.91	−0.95	−4.76
ERaptR8	CGGGUCAGGGUGACCUC	−1.78	−1.99	−0.98	−4.75
ERaptR9	UGGUCAGGCUGGUCUCA	−1.31	−1.38	−1.95	−4.63
ERaptR10	CCGGUCACGUGGGCGCG	−1.53	−1.44	−1.63	−4.60
ERaptR11	AGGGUCAUGGUGACCCU	−0.99	−1.74	−1.87	−4.59
ERaptR12	GGGGUCAAGGUCACCGC	−1.50	−1.61	−1.42	−4.52
ERaptR13	CAGGUCAAGGCGAUCUU	−1.67	−1.36	−1.23	−4.26
ERaptR14	CCAGUCAGUCUGAUUAU	−1.30	−2.02	−0.68	−4.00
ERaptR15	GCGGUCACCGUCACCUC	−1.41	−1.29	−0.76	−3.47
ERaptR16	UUGGUCAGGCUGAUCUC	−1.17	−1.84	−0.38	−3.38
ERaptR17	UUGGUCAAGCUGCCCUC	−1.33	−1.18	−0.73	−3.24
ERaptR18	GGGGUCAGCUGUGCCCCG	−1.12	−1.27	−0.51	−2.89

Docking-specific Z-scores (Z_AV_, Z_H_, and Z_P_) for individual ERα-RNA complex was calculated using the individual binding score and mean binding scores in a set of 10 best binding modes of a sequence. Total Z-score (Z_T_) was taken as sum of individual docking-specific Z-scores.

^†^The mentioned RNA sequences represent their respective complex with 1SJ0 (ERα).

**Table 3:** Predicted free energy of secondary structure formation of probable RNA aptamers and random RNA sequences.

S. No.	Sequence	Sequence name	Length (nt)	Predicted Δ*G* (kcal/mol)
1	gaggucacugagaccau	ERaptR1	17	−5.70
2	ccaggucacagugaccug	ERaptR2	18	−8.60
3	agggucagggccaccug	ERaptR3	17	−4.40
4	ggggucaaggugacccc	ERaptR4	17	−10.70
5	aaggucacgguggccac	ERaptR5	17	−5.40
6	uagcuuaucagacug	Random1	15	−0.21
7	gcugggaaacacccagg	Random2	17	−7.80
8	guugcauuuaggugcau	Random3	17	−4.30
9	cauagcagacagcuauc	Random4	17	−3.70
10	aauuuccacaggaaagca	Random5	18	−2.40

**Table 4:** Thermodynamic parameters derived from ITC for ER-RNA (ERE analogs) interactions at 25 °C^a^ ^,b^.

RNA sequences	n	*K*a (M^−1^)	Δ*H* (kcal/mol)	*T*Δ*S* (kcal/mol)	Δ*G* (kcal/mol)
ERaptR1	0.4	9.20E + 07	−109.6	−98.6	−10.96
ERaptR2	0.5	7.60E + 07	−103.6	−92.6	−10.92
ERaptR3	0.4	5.24E + 07	119.3	−108.7	−10.53
ERaptR4	0.5	1.02E + 08	−100.2	−89.1	−11.10
ERaptR5	0.4	6.82E + 07	−97.2	−86.4	−10.78
ERaptR6	0.4	9.46E + 07	−89.5	−78.6	−10.82
ERaptR7	0.5	4.82E + 07	−84.8	−74.2	−10.60
ERaptR8	0.5	8.88E + 07	−117.8	−106.9	−10.81
ERaptR9	0.5	9.46E + 07	−97.2	−86.4	−10.78
ERaptR11	0.5	8.02E + 07	−104.7	−93.8	−10.83
ERaptR12	0.4	8.20E + 07	−100.5	−86.8	−10.80
ERaptR13	0.4	8.96E + 07	−89.7	−78.9	10.73
ERaptR14	0.3	7.42E + 07	−100.6	−89.6	−10.90
ERaptR15	0.3	9.25E + 07	−140.3	−129.3	−10.96
ERaptR16	0.4	9.40E + 07	−124.7	−113.8	−10.86
ERaptR17	0.4	1.26E + 08	−99.5	−88.5	−10.99
ERaptR18	0.3	9.91E + 07	−108.3	−97.7	−10.55

^a^All parameters (n = number of binding sites, *K*a = association constant, Δ*H* = change in enthalpy, *T*Δ*S* = change in entropy, Δ*G* = Gibb’s free energy) were derived from ITC experiments conducted at 25 °C in Tris-HCl buffer (pH 8.0). Protein (ERα) concentration in cell was taken as 1 μM and RNA aptamers (R1 − R18) in syringe were taken as 10 μM. Δ*H,* Δ*S,* Δ*G* values are within 5% error and *Ka* values are within 10% error obtained from the three experimental replicates.

^b^Binding parameters for aptamer ERα-ERaptR10 couldn’t be calculated due to some unexpected irregularity in the synthesis of ERaptR10.
