# Supplementary material for: In silico selection of an aptamer to estrogen receptor alpha using computational docking employing estrogen response elements as aptamer-alike molecules
Source: Sci Rep. 2016 Feb 22;6:21285. doi: 10.1038/srep21285 (PMC4761961; doi:10.1038/srep21285)
Supplement: Supplementary Information [file srep21285-s1.doc]

# Supplementary Information

***In silico* selection of an aptamer to estrogen receptor alpha using computational docking employing estrogen response elements as aptamer-alike molecules**

Rajesh Ahirwar, Smita Nahar,Shikha Aggarwal, Srinivasan Ramachandran, Souvik Maiti and Pradip Nahar

**Preparation of nuclear and cytoplasmic extracts**

MCF-7 and MDA MB-231 cells were maintained in T-75 flasks in high glucose DMEM (Gibco, Life Technologies) with 100 IU/ml Penicillin, 100 µg/ml Streptomycin and 0.25 µg/ml Amphotericin B (Cell Clone, Genetix) supplemented with 10% FBS. For nuclear extract preparation, the cells of a T-75 flask were first collected and resuspended in 500 μL of 1X hypotonic solution. After 15 min of incubation on ice, 25 μL of 10%NP-40 detergent was added to these cells and vortexed vigirously. The cells were centrifuge immediately at 3000 rpm at 4°C. The supernatant was collected as cytosolic extract while the pelleted was dissolved in 50 μL cell extraction buffer (ThermoFisher Scientific, USA). After 30 min of incubation on ice, the solution was centrifuged at 10,000 g for 30 min and the supernated was collected as the nuclear extract.

**Tables 1.** Control set of docking on thrombin-RNA aptamer and VEGF165-RNA aptamer complexes. Random sequences of the same length as that of respective aptamers were used to ascertain the selectivity in binding predictions.

| Ligand name | Sequences | Length (nt) | Target | AutoDock Vina Z-score (Z_AV_) | PatchDock Z-score (Z_P_) | Haddock Z-score (Z_H_) | Total Z-score (Z_T_) |
| --- | --- | --- | --- | --- | --- | --- | --- |
| Thrombin aptamer | gggaacaaagcugaaguacuuaccc | 25 | 1PPB | -1.9 | -1.4 | -2.3 | -5.6 |
| T-Random 1 | cggaauuauauucauaccuccgggg | 25 | 1PPB | -1.3 | -1.3 | -1.3 | -3.9 |
| T-Random 2 | uacggaccgguauacucucaguuga | 25 | 1PPB | -0.7 | -1.1 | -1.4 | -3.3 |
| T-Random 3 | cauuugagacugcuaugucagacgc | 25 | 1PPB | -0.7 | -1.1 | -1.4 | -3.3 |
| T-Random 4 | cggcuauuauggaaaaucuucccgg | 25 | 1PPB | -0.6 | -1.6 | -1.5 | -3.8 |
| T-Random 5 | uuuaggacaucccaugucagcggau | 25 | 1PPB | -1.5 | -0.6 | -1.8 | -3.8 |
| VEGF_165_ aptamer | ccgguagucgcauggcccaucgcgcccgg | 29 | 2VGH | -1.5 | -1.4 | -2.1 | -5.0 |
| V-Random 1 | guaaugucuuaaauagcguaaaacaguaa | 29 | 2VGH | -0.9 | -1.2 | -1.4 | -3.5 |
| V-Random 2 | uuccuguugcauucguaccgccuauauuu | 29 | 2VGH | -0.8 | -0.6 | -2.0 | -3.4 |
| V-Random 3 | gucucuuugccggcuuauauggacaagca | 29 | 2VGH | -1.1 | -0.7 | -1.6 | -3.4 |
| V-Random 4 | uaacauaacuauuuaucgaaauaccuucg | 29 | 2VGH | -1.1 | -0.6 | -1.3 | -3.0 |
| V-Random 5 | gaaugaaaucauuuaauuuugugccguag | 29 | 2VGH | -1.3 | -1.0 | -1.4 | -3.7 |

**Table 2.** Control docking of ERα (1SJ0) with stable hairpins forming random RNA sequences.

| S. No. | Random RNA ligands for ER (form stable hairpin loops) | Length (nt) | AutoDock Vina Z-score (Z_AV_) | PatchDock Z-score (Z_P_) | Haddock Z-score (Z_H_) | Total Z-score (Z_T_) |
| --- | --- | --- | --- | --- | --- | --- |
| 1 | uagcuuaucagacug | 15 | -1.0 | -1.43 | -0.90 | -3.34 |
| 2 | gcugggaaacacccagg | 17 | -1.2 | -1.41 | -1.51 | -4.21 |
| 3 | guugcauuuaggugcau | 17 | -0.8 | -0.85 | -1.19 | -2.84 |
| 4 | cauagcagacagcuauc | 17 | -1.6 | -0.49 | -1.52 | -3.60 |
| 5 | aauuuccacaggaaagca | 18 | -1.1 | -1.27 | -1.48 | -3.85 |

**Table 3.** List of H-bonding residues in the docking predicted ER complex of ERα-ERaptR4 complex^a^.

| Donor atom | Chain ID | Residue number | Interacting atom | Acceptor atom | Chain ID | Residue number | Interacting atom | H-bond distance |
| --- | --- | --- | --- | --- | --- | --- | --- | --- |
| **Haddock** |  |  |  |  |  |  |  |  |
| ARG | A | 434 | NH2 | ADE | B | 13 | O2P | 2.95 |
| HIS | A | 501 | NE2 | CYT | B | 16 | O1P | 2.89 |
| GLN | A | 502 | NE2 | CYT | B | 15 | O1P | 2.91 |
| **PatchDock** |  |  |  |  |  |  |  |  |
| TRP | A | 383 | NE1 | GUA | A | 2 | O3' | 2.04 |
| TYR | A | 526 | OH | GUA | A | 2 | O2' | 2.13 |
| TYR | A | 526 | OH | GUA | A | 3 | O4' | 2.38 |
| SER | A | 527 | N | CYT | A | 17 | O2' | 2.96 |
| CYT | A | 15 | N4 | VAL | A | 533 | O | 2.93 |
| CYT | A | 16 | N4 | VAL | A | 533 | O | 2.44 |
| LEU | A | 536 | N | GUA | A | 3 | N7 | 2.77 |
| ADE | A | 13 | N6 | ASP | A | 538 | OD2 | 2.88 |
| **AutoDock Vina** |  |  |  |  |  |  |  |  |
| GUA | A | 1 | N2 | ASP | A | 321 | OD2 | 2.85 |
| GLY | A | 366 | N | GUA | A | 2 | O6 | 2.80 |
| GUA | A | 12 | O2' | ASP | A | 369 | O | 2.72 |
| ARG | A | 477 | NH2 | ADE | A | 13 | O2' | 2.90 |
| GUA | A | 3 | O2' | HIS | A | 547 | O | 2.98 |
| GUA | A | 3 | H22 | MET | A | 543 | O | 2.29 |
| GUA | A | 12 | HO2' | ASP | A | 369 | O | 1.93 |
| ALA | A | 307 | N | ADE | A | 7 | N1 | 3.18 |
| ASN | A | 359 | ND2 | GUA | A | 3 | O6 | 3.19 |
| ARG | A | 363 | NE | GUA | A | 2 | OP2 | 3.03 |
| ARG | A | 548 | NE | CYT | A | 17 | O2 | 3.07 |
| ARG | A | 548 | NH1 | GUA | A | 4 | O5’ | 3.13 |
| ARG | A | 548 | NH2 | URI | A | 11 | OP2 | 3.11 |

^a^Atoms involved in the H-bond formation are represented as donors and acceptors. Molecule representation follows a three letter code preceded by its chain ID, residue number and interacting atom. Distance of H-bond is in angstrom (Å).


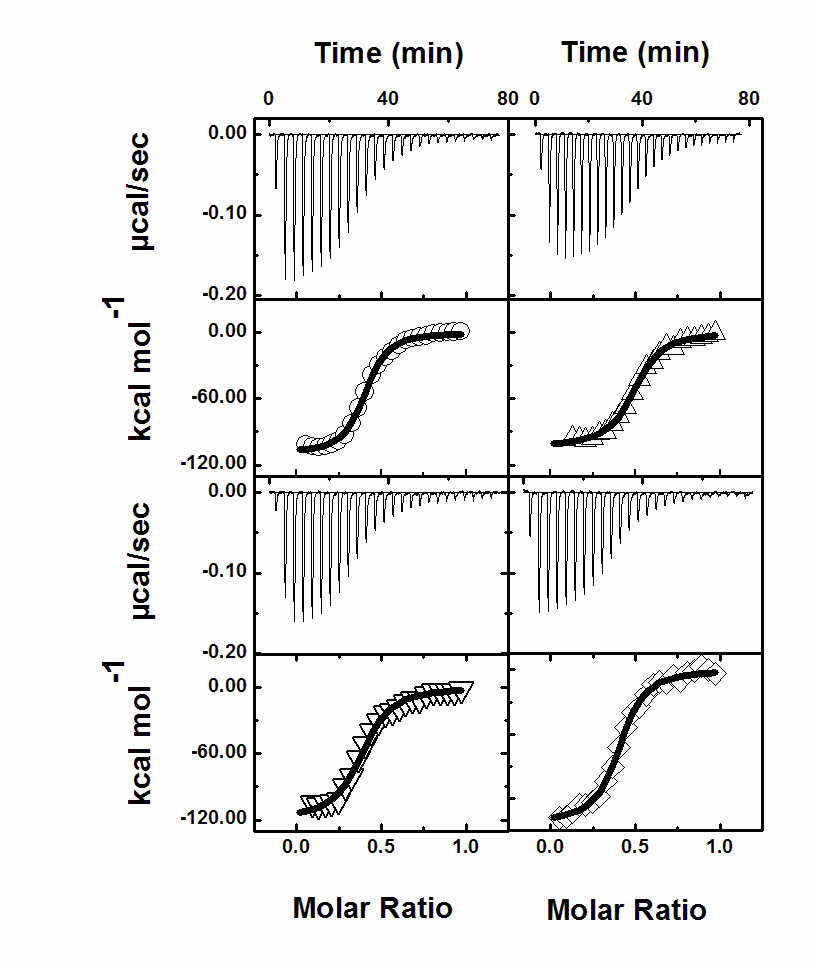


**Figure 1**. ITC isotherms of ERα interactions with aptamers, ERaptR1 (O), ERaptR2 (∆),ERaptR3 (∇) and ERaptR5 (◊). For each titration, the ERα concentration in 1.4 ml sample cell was taken as 1μM and aptamer concentration in syringe was 10 μM. The top panel represents the raw heats of binding obtained upon titration of aptamer to ERα protein. The lower panel is the binding isotherm fitted to the raw data using one site model.


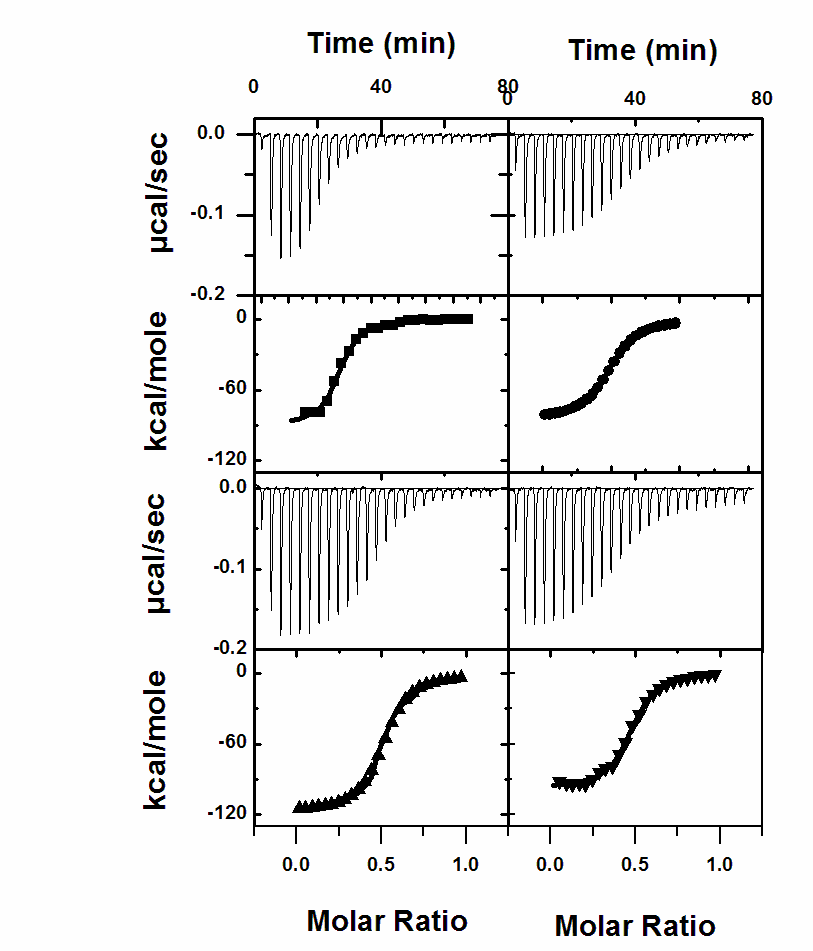


**Figure 2**. ITC isotherms of ERα interactions with aptamers, ERaptR6 (■),ERaptR7 (●),ERaptR8 (▲) and ERaptR9 (▼). For each titration, the ERα concentration in 1.4 ml sample cell was taken as 1μM and aptamer concentration in syringe was 10 μM. The top panel depicts the raw heats obtained upon titration of aptamer to ERα protein. The lower panel is the binding isotherm fitted to the raw data using one site model.


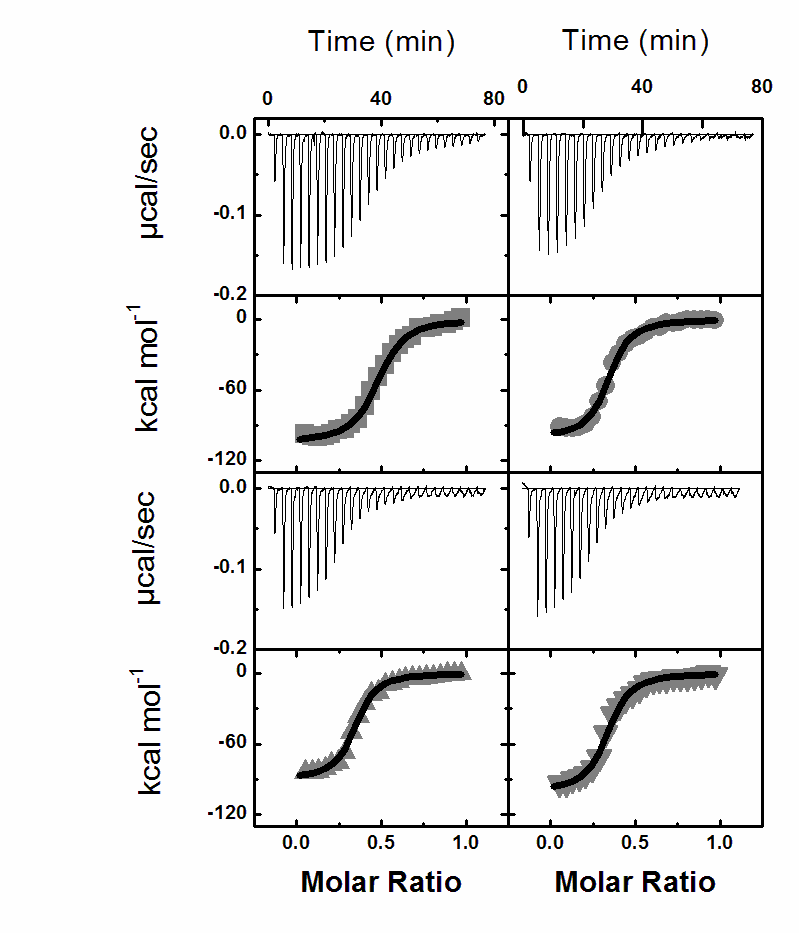


**Figure 3.** ITC isotherms of ERα interactions with aptamers, ERaptR11 (■),ERaptR12 (●),ERaptR13 (▲) and ERaptR14 (▼). For each titration, the ERα concentration in 1.4 ml sample cell was taken as 1μM and aptamer concentration in syringe was 10 μM. The top panel depicts the raw heats obtained upon titration of aptamer to ERα protein. The lower panel is the binding isotherm fitted to the raw data using one site model.


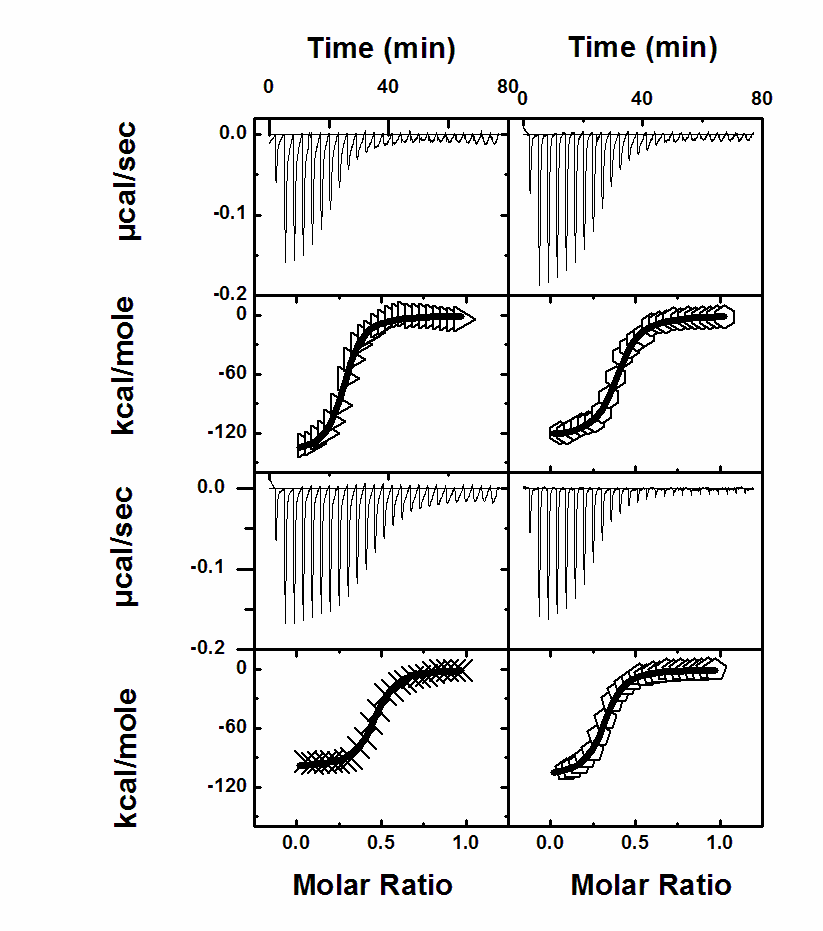


**Figure 4.** ITC isotherms of ERα interactions with aptamers, ERaptR15 (∇),ERaptR16 (◊),ERaptR17 (×) and ERaptR18 (⌂). For each titration, the ERα concentration in 1.4 ml sample cell was taken as 1μM and aptamer concentration in syringe was 10 μM. The top panel represents the raw heats obtained upon titration of aptamer to ERα protein. The lower panel is the binding isotherm fitted to the raw data using one site model.
